# Quantification and Classification of *E*. *coli* Proteome Utilization and Unused Protein Costs across Environments

**DOI:** 10.1371/journal.pcbi.1004998

**Published:** 2016-06-28

**Authors:** Edward J. O’Brien, Jose Utrilla, Bernhard O. Palsson

**Affiliations:** 1 Department of Bioengineering, University of California, San Diego, La Jolla, California, United States of America; 2 Bioinformatics and Systems Biology program, University of California, San Diego, La Jolla, California, United States of America; 3 Centro de Ciencias Genómicas. Universidad Nacional Autónoma de México, Cuernavaca, Morelos, México; 4 Novo Nordisk Foundation Center for Biosustainability, Technical University of Denmark, Lyngby, Denmark; 5 Department of Pediatrics, University of California, San Diego, La Jolla, California, United States of America; The Pennsylvania State University, UNITED STATES

## Abstract

The costs and benefits of protein expression are balanced through evolution. Expression of un-utilized protein (that have no benefits in the current environment) incurs a quantifiable fitness costs on cellular growth rates; however, the magnitude and variability of un-utilized protein expression in natural settings is unknown, largely due to the challenge in determining environment-specific proteome utilization. We address this challenge using absolute and global proteomics data combined with a recently developed genome-scale model of *Escherichia coli* that computes the environment-specific cost and utility of the proteome on a per gene basis. We show that nearly half of the proteome mass is unused in certain environments and accounting for the cost of this unused protein expression explains >95% of the variance in growth rates of *Escherichia coli* across 16 distinct environments. Furthermore, reduction in unused protein expression is shown to be a common mechanism to increase cellular growth rates in adaptive evolution experiments. Classification of the unused protein reveals that the unused protein encodes several nutrient- and stress- preparedness functions, which may convey fitness benefits in varying environments. Thus, unused protein expression is the source of large and pervasive fitness costs that may provide the benefit of hedging against environmental change.

## Author Summary

An overarching endeavor in systems biology is to characterize and understand the allocation of an organism’s proteome. Common approaches to characterize proteome allocation are based on annotations of protein functions or transcriptional regulatory targets. Here, we develop a novel approach based on model-predicted proteome utilization. This approach reveals that in many environments, a large fraction of the proteome is unused. Unused protein expression is known to incur costs on organismal fitness. We show that changes in the allocation of the proteome to used versus unused fractions can account for the variability in growth rates observed across environments and is a common mechanism to increase growth rates in laboratory evolution experiments. We compare our approach to classify the proteome based on model-predicted utilization to more traditional approaches to reveal biological functions and transcriptional regulators underlying the expression of unused protein. Expression of these functions may reflect ecological trade-offs between growth, nutrient-readiness, and stress resistance.

## Introduction

The costs and benefits of protein synthesis on organismal fitness shape the evolution and regulation of proteome expression [1]. For microbes, the rate of cellular growth is an important component of organismal fitness [2], and much of the microbial proteome is devoted to growth in certain environments [3].

Protein costs and benefits are often quantified by their effects on cellular growth [1,4]. Notably, the synthetic overexpression of unused protein results in a predictable linear reduction in growth rates [4,5]. Microbes will express some amount of unused protein in any given environment, which will reduce their growth rates [6]. However, the magnitude and variability of unused protein expression is unknown, largely due to the challenge in determining environment-specific proteome activity. Thus, we do not yet have a baseline understanding of the percentage of the microbial proteome that is un-utilized in a given environment or how much the un-utilized protein fraction can vary (or if it remains predominantly constant across environments).

Here, we combine a recently developed genome-scale model of proteome allocation in *Escherichia coli* [7] with genome-scale absolute proteomics measurements to quantify proteome allocation across 16 distinct environments [8]. This approach allows us to enumerate the unused proteome, revealing that the unused protein fraction can vary greatly across environment. We subsequently quantify the cost of unused protein expression and characterize the processes and regulators underlying the change in unused protein expression across environments. In turn, we show that unused protein expression is the source of large and pervasive fitness costs that may provide the benefit of hedging against environmental change.

## Results

### Defining the un-utilized and under-utilized ME proteome

**Fig 1:**
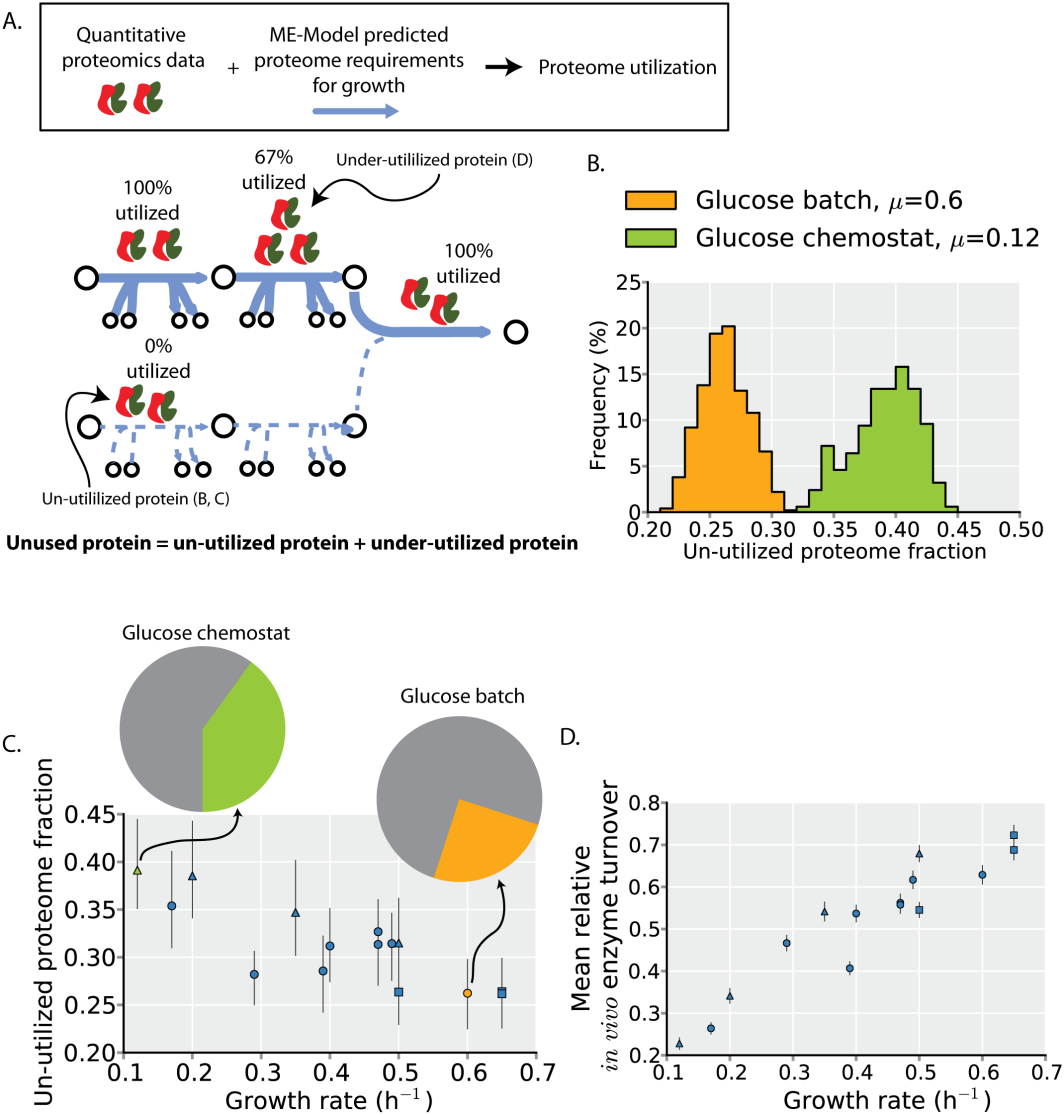
Unused protein abundances are not constant across environments. (**A**) Graphical illustration of the two classes of unused protein. The first class is protein that is expressed but completely un-utilized (i.e., 0% utilized) first step in lower pathway). The second class is protein that is under-utilized (i.e., <100% but >0% utilized; second step in upper pathway, see Methods). The percent utilization indicated is based on the upper pathway having flux of 2, the lower pathway having flux of 0, and all enzymes having a maximal rate of 1 (see also S1 Fig). (**B**) The un-utilized proteome fractions for batch and chemostat culture on glucose minimal media. The un-utilized proteome fraction is a distribution rather than a specific value because of different potential alternative pathways and enzymes that can be used to support cellular growth (see main text). (**C**) The un-utilized proteome fraction across all profiled environments—8 different carbon source batch cultures (circles), 4 glucose-limited chemostat cultures (triangles), and 4 stress conditions (squares)—is plotted as a function of the growth rate measured in that condition. Error bars indicate 2.5 and 97.5 percentiles of the un-utilized proteome distributions for each condition (e.g. Fig 1B). The orange and green points and pie charts correspond to the environments in B of the same color. (**D**) Enzyme turnover rates tend to increase at higher growth rates. Changes in turnover rates indicate under-utilized protein. The mean relative turnover (across all proteins) is plotted with error bars indicating the 95% confidence interval for the mean (the full distribution is shown in S1 Fig). Point shape indicates environment type as in C.

We distinguish between two classes of unused protein (Fig 1A and S1 Fig). The first class is the *un-utilized* protein. This is protein that, in the specified environment, is not utilized for cellular growth. For example, in glucose minimal media, the glycerol transporter is un-utilized, but it might be expressed. The second class of unused protein is the *under-utilized* protein. This is protein that is catalytically active, but is present in excess and is thus operating under its maximal capacity.

We use a genome-scale model of proteome allocation in *Escherichia coli*, termed a ME-Model [7,9] to quantify un- and under-utilized protein. The ME-Model formalizes the function and synthesis of all proteins that carry out metabolism and protein expression necessary for growth, which we refer to as the ME proteome. The ME proteome encompasses much of the proteome required for cellular growth and accounts for ~80% of the proteome by mass in conditions of exponential growth [7].

From ME-Model simulations we identify proteins that are needed for growth in a particular environment. By combining these simulations with quantitative proteomics data, we identify proteome-wide changes in both un- and under-utilized protein abundances (see Methods). In a given environment, there are several different alternative pathways and enzymes can be used to support cellular growth [10–12]. Thus, the computed un-utilized proteome fraction needs to be assessed across all such alternate growth states; randomized sampling of the alternate network states accomplishes this and leads to a distribution of possible un-utilized proteome fractions rather than a single specific value (Fig 1B, S1 Fig, see Methods). To compute changes in under-utilized protein expression, we identify relative changes in protein catalytic activity across environments. As the ratio of model-predicted protein demands for biosynthesis and measured protein expression levels is proportional to *in vivo* enzyme turnover rates, changes in this ratio are proportional to *in vivo* enzyme turnover rates and are used to quantify under-utilized protein (see Methods).

### Un-utilized and under-utilized ME proteome abundance varies across environments

We first compare the un-utilized proteome fraction during growth in glucose batch culture and chemostat culture. As the nutrient source is the same in these two environments, the set of proteins that can be utilized for growth in the ME-Model are identical; variation in the proteomics data will determine differences in the un-utilized proteome abundances. As the two distributions of un-utilized proteome abundance are non-overlapping, the un-utilized proteome fraction does vary significantly between these two environments (Fig 1B).

When data from multiple growth conditions (8 different carbon sources in batch cultures, 4 glucose-limited chemostat cultures, and 4 stress conditions—acid, osmotic, temperature, and aerobicity; see Methods) are analyzed in a similar manner, a clear general trend emerges in which environmental conditions resulting in higher growth rates tend to have lower un-utilized proteome fractions (Fig 1C). This correlation suggests that un-utilized proteome fraction is an important source of growth rate variation.

Next we consider the under-utilized proteome under the same growth conditions by computing the variation in *in vivo* enzyme turnover (flux per protein; see Methods). We find that the abundance of the under-utilized proteome is not constant across environments; rather, the average enzyme turnover tends to increase in environments with higher growth rates (Fig 1D). This trend has been observed for several individual proteins using lower throughput methods [7,13,14].

Thus, we find that the amount of both un-utilized protein and under-utilized protein is reduced with increasing cellular growth rate.

### Growth rate variation can be explained by unused proteome expression

**Fig 2:**
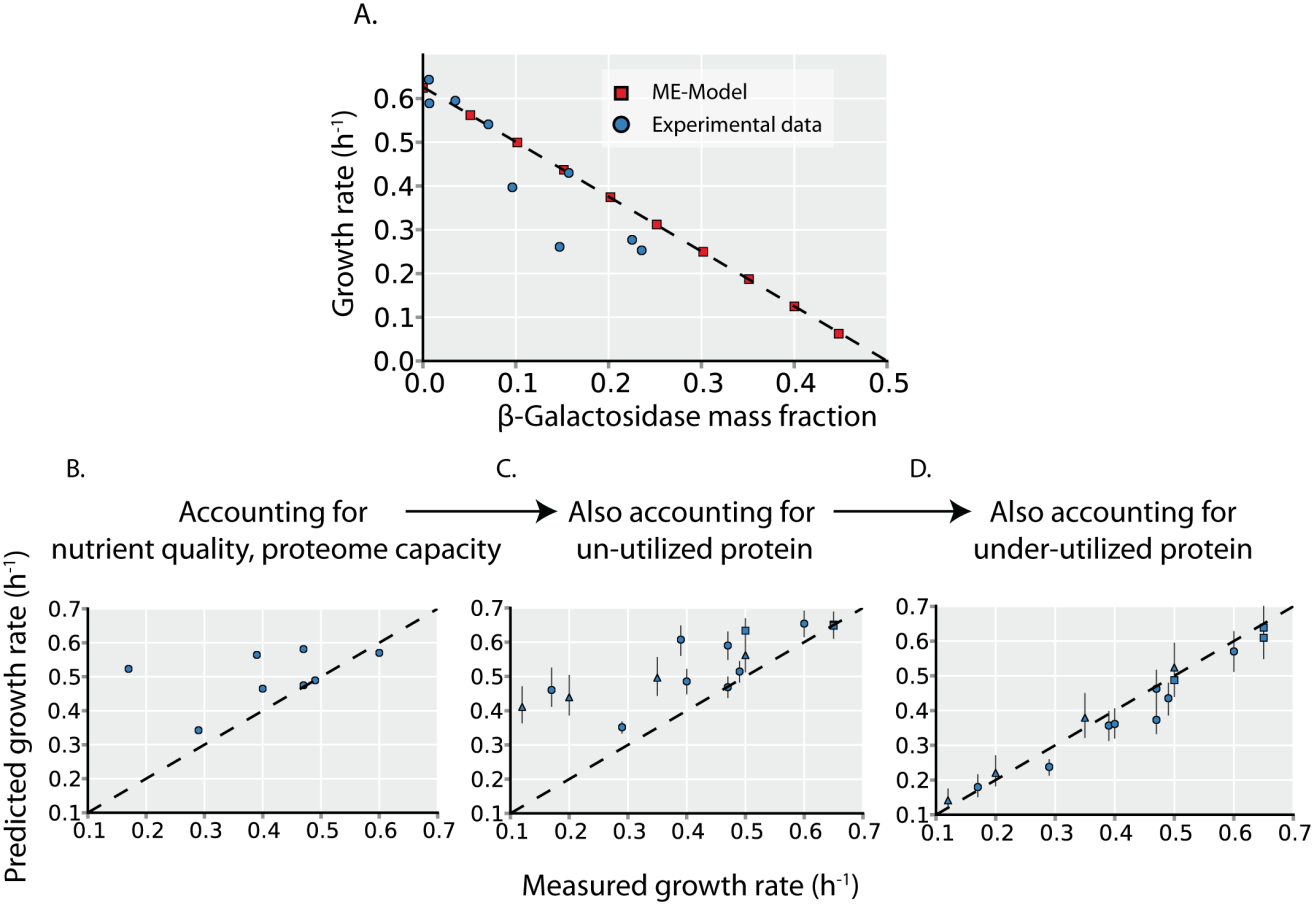
Growth rate is determined by unused protein expression. (**A**) To validate that the ME-Model accurately quantifies protein cost, we compare the predicted effect of un-utilized protein (β-Galactosidase) overexpression (red squares) to experimental measurements (blue circles). Experimental data is obtained from Scott et al. [4] The x- and y- intercept from a linear regression (dotted line) is consistent with the phenomenological model of Scott et al. [4] The effects of the un-utilized and under-utilized protein expression (Fig 1) on growth rates are quantified in C and D. (**B**) Predicted growth rates are plotted versus measured growth rates during batch growth on 8 different carbon sources. Predicted growth rates are the computed maximal growth rates by the ME-Model, assuming the un-utilized proteome fraction and *in vivo* turnover rates are the same across all environments (see Methods, this assumption is eliminated in panels B and C to assess the effects of un- and under-utilized protein on growth). (**C**) Predicted maximal growth rates are computed with the ME-Model with the un-utilized proteome fraction set to the values inferred from proteomics data (see Methods and Fig 1C). In addition to the 8 carbon sources (circles), 4 glucose-limited chemostat cultures (triangles) and 4 stress conditions (squares) are also shown. These additional growth conditions are not included in panel A as growth rate predictions would require further information. (**D**) Predicted maximal growth rates are computed with the ME-Model with both the un-utilized proteome fraction and the *in vivo* turnover rate (indicative of under-utilized protein, Fig 1D) set to the values inferred from proteomics data. Point shape indicates environment type as in B (carbon source batch cultures = circles, glucose-limited chemostat cultures = triangles, stress conditions = squares).

Un-utilized proteome fraction and *in vivo* enzyme turnover are variables that are formalized in the ME-Model. As the ME-Model accurately predicts the growth effects of unused protein expression (Fig 2A and S2 Fig), setting these parameters to measured values can determine how they quantitatively affect growth rates.

As a baseline for comparison, we first predict maximum growth rates across different carbon sources with the ME-Model while holding the un-utilized proteome fraction and *in vivo* enzyme turnover rates constant across environments; this assumption allows us to assess the growth rate variation due to changes in carbon sources independent of changes in un- and under-utilized protein. Comparing predicted and measured growth rates across the 8 carbon sources shows that, in general, the predictions are poor. Some particular carbon sources are well predicted (e.g. glucose, fumarate, succinate) but the correlation between predicted and measured growth rates is low (Pearson’s r = 0.39); furthermore, the variation in growth rates across the experimental data is much larger than that across the computational data (s = 0.16 compared to s = 0.7; s is the sample standard deviation). Thus, there are significant contributors to growth rate variation other than the necessary changes in metabolic pathway and proteome usage when the primary carbon source for growth is varied.

We next sequentially assess the contribution of changes in un- and under-utilized protein to growth rate variation. In addition to the 8 carbon sources (circles), we can also assess the 4 carbon-limitation (triangles) and 4 environmental stress (squares) conditions that were proteomically profiled. In these additional environments, glucose is the carbon source, and other environmental changes affect proteome expression and growth. Accounting for un-utilized protein increases the correlation between predicted and measured growth rates to r = 0.82 and increases the predicted growth rate variance to s = 0.09. Finally, accounting for the variation in *in vivo* turnover rates increases the correlation to r = 0.98 and results in a predicted growth rate variance that is similar to the experimental variance (s = 0.15).

Thus, accounting for changes in the un- and under-utilized proteome explains much of the variation in growth rates across environments (Fig 2C and 2D).

### Selection for faster growth rates results in a decrease in unused protein expression

If the expression of unused (i.e., un-utilized and under-utilized; see Fig 1A) protein is a dominant source of variation in growth rates, one would expect that a common mechanism to increase growth rates through evolution would be to down-regulate expression of unused protein.

**Fig 3:**
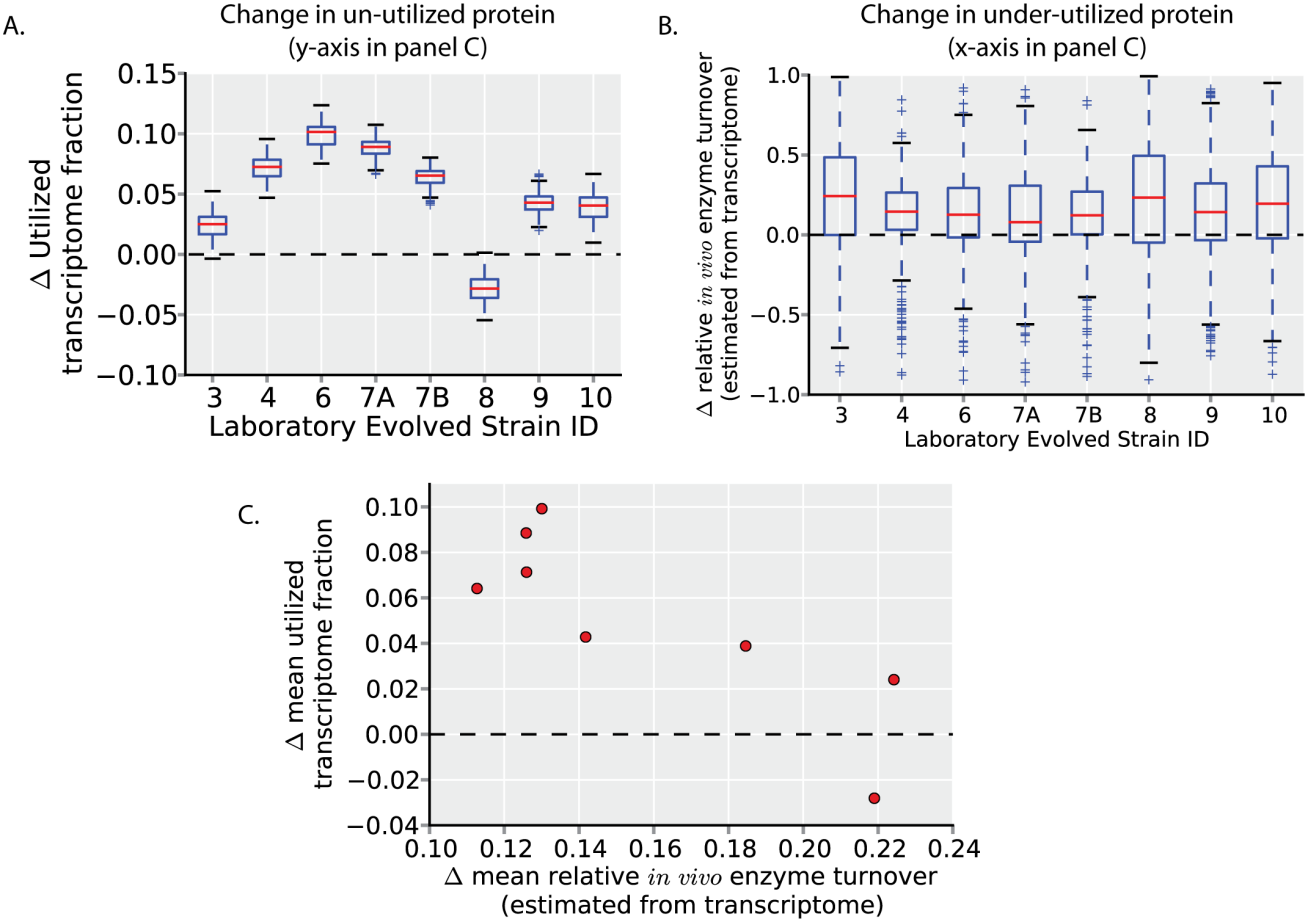
Growth rate increases through changes in unused protein allocation in adaptive evolution experiments. (**A**) The change in utilized transcriptome fraction of strains independently evolved in glucose minimal media [15] (which reached growth rates of ~1.0 h^-1^, compared to the wild-type growth rate of 0.7 h^-1^) is calculated (see Methods). All strains except one (strain 8) show an increase in the utilized transcriptome fraction (i.e., a decrease in un-utilized transcriptome). (**B**) The estimated changes in relative enzyme turnover rates compared to the wild-type (unevolved) strain are calculated (as in Fig 1D and S1 Fig; see Methods). The distributions of all strains have a median value greater than zero, indicating a decrease in under-utilized protein in evolved strains. (**C**) The mean value of the estimated change in utilized protein (y-axis, from A) is plotted versus the mean value of the estimated change in under-utilized protein (quantified by change in relative *in vivo* enzyme turnover rates; y-axis, from B). The change in utilized transcriptome is anti-correlated with the change in *in vivo* enzyme turnover, indicating that the independently evolved strains use different strategies (i.e., a decrease in un-utilized versus a decrease in under-utilized protein) to increase growth rates.

To assess this hypothesis, we estimate changes in un-utilized and under-utilized protein in *E*. *coli* experimentally evolved under a growth rate selection pressure in glucose minimal media batch culture [15]. Though it is unused protein (and protein cost) that is important for constraining growth rates, we estimate changes in unused protein with available transcriptomics data [15]. Using the same ME-Model simulations used to define un-utilized protein in glucose minimal media (Fig 1B and 1C), we calculate changes in the un-utilized transcriptome fraction after evolution (Fig 3A). All strains except one (strain 8) increase their utilized transcriptome fraction (decreasing their un-utilized transcriptome). We next calculate changes in under-utilized protein after evolution (again, using the same ME-Model simulations as in Fig 1D) by estimating changes in relative *in vivo* enzyme turnover (flux per protein). (Estimating these values with transcriptomics data assumes that, for a given protein, the translated protein per transcript is the same across the strains assessed). All strains tend to increase relative *in vivo* enzyme turnover (Fig 3B; indicated by the distributions being shifted above zero). Thus, a decrease in un-utilized and under-utilized protein expression is a common mechanism of growth rate increase in these experimentally evolved strains.

While these evolved strains tend to decrease their unused protein expression, un-utilized and under-utilized protein expression does not change uniformly across strains. Rather, across strains, the decrease in un-utilized protein expression (increase in utilized protein expression) is anti-correlated with the decrease in under-utilized protein expression (Fig 3C; increase in relative *in vivo* enzyme turnover). While each of these strains reaches approximately the same growth rate of ~1.0 h^-1^ after evolution, they seem to employ different strategies to increase their growth rates. Notably, strain 8, which decreases its utilized transcriptome expression (which would tend to decrease, rather than increase growth rates), has among the highest increases in relative *in vivo* enzyme turnover. Strain 8 also decreases its ribosomal protein expression compared to wild-type, despite the higher translational demands of faster growth rates (S3 Fig); this suggests higher relative *in vivo* enzyme turnover rates of ribosomal proteins (amino acid per ribosome per second) in this strain.

Therefore, the observed changes in gene expression after selection for faster growth rates indicates that decreasing un-utilized and under-utilized protein expression are common mechanisms to increase growth rates. These mechanisms are consistent with the expression changes observed in other evolved strains [16] and identified causal mutations [17,18]. Even though glucose minimal media batch culture is an environment with among the lowest unused protein expression levels across the environments profiled with proteomics (Fig 1), unused protein expression is still substantial and decreases after adaptive evolution.

### Model-driven classification of proteome segments

Why does *E*. *coli* allocate its proteome in a way that detracts from achieving its maximal growth rate? And what regulatory processes underlie this behavior? To answer these questions, we first systematically segment the proteome by protein function. We will then be able to attribute changes in un-utilized and under-utilized protein to specific functional proteome segments. We segment the ME proteome by classifying each protein by its environment-specific utility through growth simulations with the ME-Model under all Carbon, Nitrogen, Phosphorous, and Sulfur sources; this results in many environment-specific growth-supporting proteomes.

**Fig 4:**
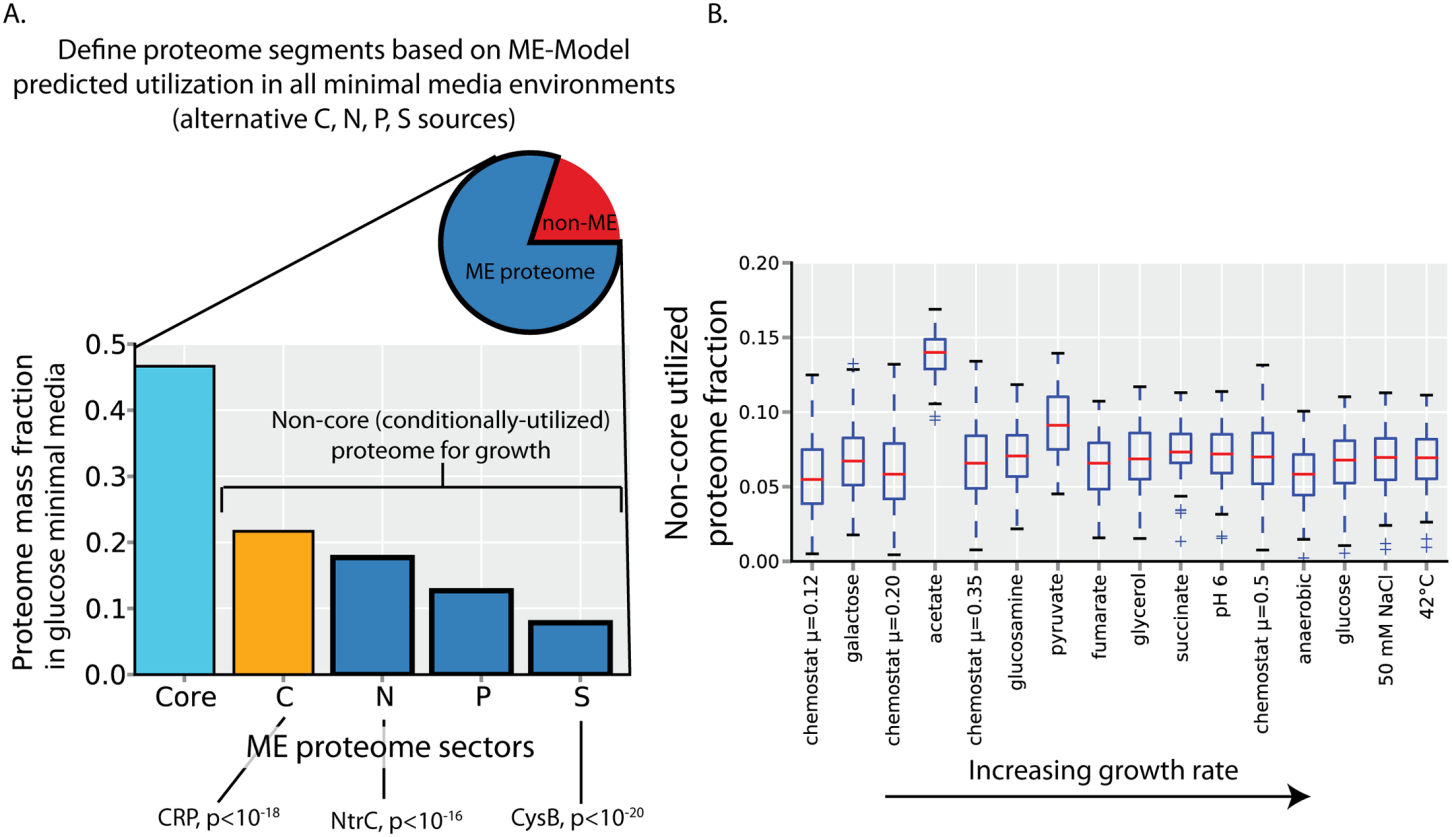
Definition and utilization of the ME proteome functional segments. (**A**) The ME proteome encompasses ~80% of the proteome by mass in glucose minimal media (pie chart). ME-Model growth simulations are used to define proteome segments (see Methods). The core proteome is composed of proteins that are used in all minimal media environments simulated. The C-, N-, P-, S-proteome segments contain proteins that are expressed under at least one alternative carbon, nitrogen, phosphorous or sulfur source environments. Abundance of these proteome segments in glucose minimal media batch culture is shown. Several global transcription factors have targets that are highly enriched (p<10^−15^) in the segments shown. (**B**) In a particular environment, the utilized proteome is composed of the core proteome and some of the non-core (condition-specific) proteome. Shown is the fraction of the proteome that is utilized and in the non-core proteome. Boxplots indicate the range of potential values resulting from sampling of model enzyme activity values (see Methods). Conditions are ordered by increasing growth rates. Other than growth on acetate, the fraction proteome that is non-core and utilized remains relatively constant. Compared to the core proteome mass fraction (which is utilized under all environments by definition), the non-core proteome comprises a relatively small proportion of the utilized proteome.

Comparing the environment-specific growth-supporting proteomes reveals a common ‘core’ proteome that is utilized across all (minimal media) environments, as previously detailed in Yang et al. [19] (Fig 4A, S1 Table). This core proteome is largely involved in anabolism and protein synthesis (including the necessary transcription, translation, and protein folding enzymes; S4 Fig).

In addition to the core proteome, there are non-core (i.e., conditionally-utilized) proteins that we classify by element source into the C-proteome, N-proteome, P-proteome, and S-proteome (that are used for growth under alternative Carbon, Nitrogen, Phosphorous, and Sulfur sources, respectively) (Fig 4A, S1 Table). These proteome segments are largely catabolic S4 Fig). Several of the non-core proteome segments show a large enrichment of transcriptional regulatory targets. The C-proteome is regulated by CRP, the N-proteome by NtrC, and the S-proteome by CysB (Fig 4A). These global transcription factors are known to respond to changes in the availability of the corresponding nutrient sources [20–22], supporting the model-driven definition of these proteome segments.

The core proteome is utilized across all environments (by definition), but in any given environment, a portion of the non-core (conditionally-utilized) proteome will also be utilized. We found that an approximately constant ~6% of the proteome is non-core and utilized across the environments profiled with proteomics (other than growth on acetate as a carbon source as an outlier). Thus, across the environments profiled with quantitative proteomics, the non-core proteome is largely un-utilized, and the core proteome constitutes the vast majority of the utilized proteome (Fig 4B). In the following sections, we detail the regulation of the core and non-core proteome segments to relate changes in these proteome segments to the observed changes in un-utilized and under-utilized protein (Fig 1).

### The core ME proteome is under-utilized

The core proteome abundance increases linearly with growth rate, consistent with higher biosynthetic demands at faster growth rates. This linear relation with growth rate has been observed for individual proteins within the core proteome [14], and serves to further validate the model-based definition of the core proteome [19].

**Fig 5:**
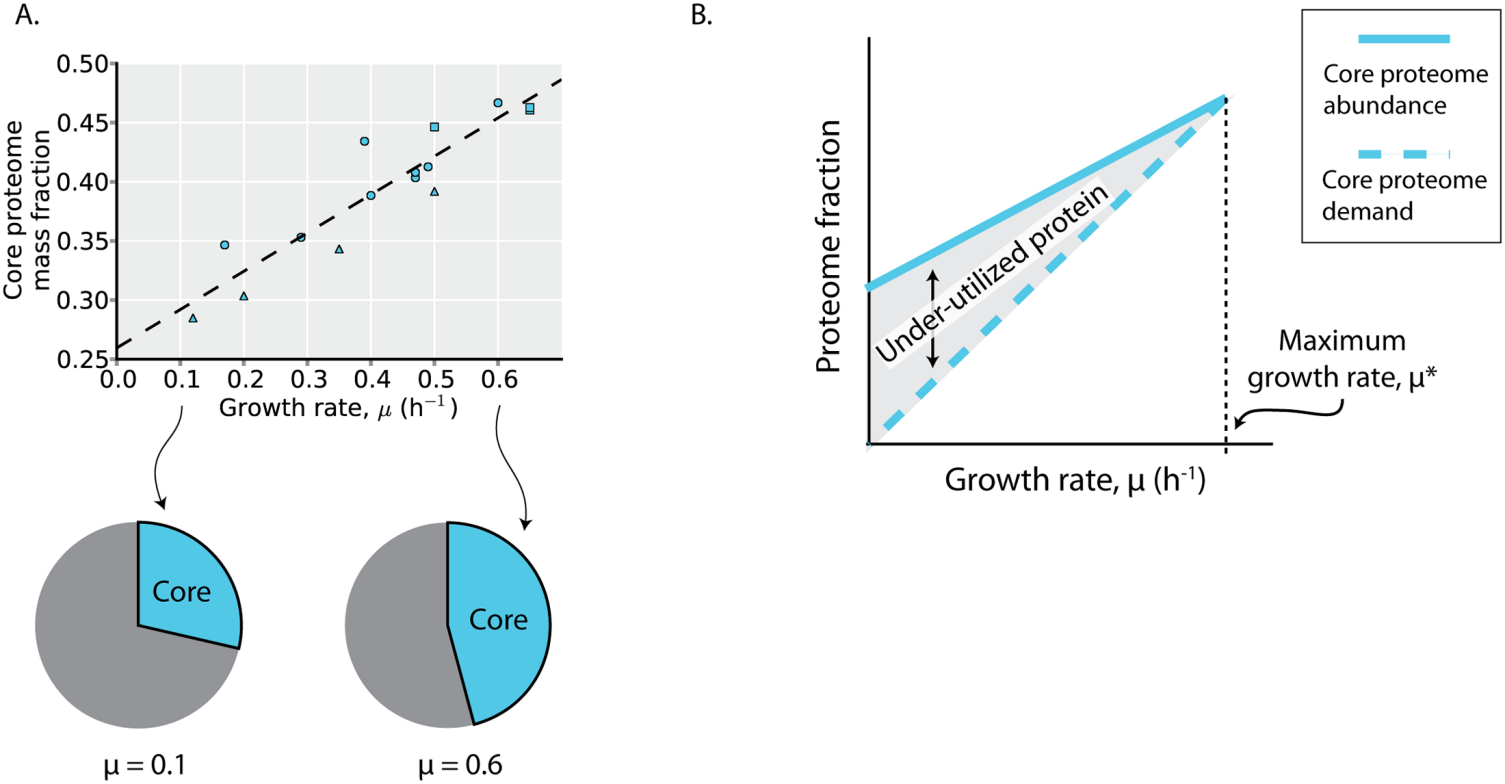
The core proteome is under-utilized. (**A**) The core proteome mass fraction plotted as a function of growth rate in the profiled environments. The dashed line is a linear regression (y = 0.33 x + 0.26, r^2^ = 0.85, p < 10^−5^). Point shape indicates environment type (carbon source batch cultures = circles, glucose-limited chemostat cultures = triangles, stress conditions = squares). (**B**) Depicted is a depiction of how the expression trends of the core proteome result in under-utilized protein. The core proteome abundance (solid line) is expressed at a level above its demands for growth (dashed line) at lower growth rates, resulting in under-utilized protein and a growth rate-dependent change in *in vivo* enzyme activity (Fig 1D). These growth rate-dependent changes have been experimentally demonstrated for ribosomal proteins [13,14].

While the core proteome is (defined to be) utilized under all environments examined, enzymes in the core proteome may vary in their *in vivo* rate of catalysis across environments (e.g., due to changes in the concentrations of substrates, products, or allosteric regulators), as observed in Fig 1D. While the core proteome abundance increases linearly with growth rate, it is over-expressed compared to its biosynthetic demands, as evidenced by the non-zero y-intercept in Fig 5A. This overexpression of proteins in the core proteome was previously observed for ribosomal proteins, leading to the realization that translation rates are growth rate dependent: the catalytic rate of ribosomes (amino acids per second per ribosome) increases with higher growth rates, approaching its maximal catalytic rate at the *E*. *coli’s* maximal growth rate [7,13,14]. A similar trend appears to be true for the core proteome more generally (Fig 5B). At higher growth rates, the core proteome abundance approaches its demand, resulting in an increase in *in vivo* enzyme turnover at higher growth rates; at lower growth rates, the core proteome is under-utilized. As the core proteome comprises the majority of the utilized protein across the (minimal media) environments profiled with proteomics (Fig 4B), over-expression of the core proteome underlies much of the observed changes in under-utilized protein across environments (Fig 1D).

### The non-core ME proteome is largely un-utilized in any particular minimal media environment

**Fig 6:**
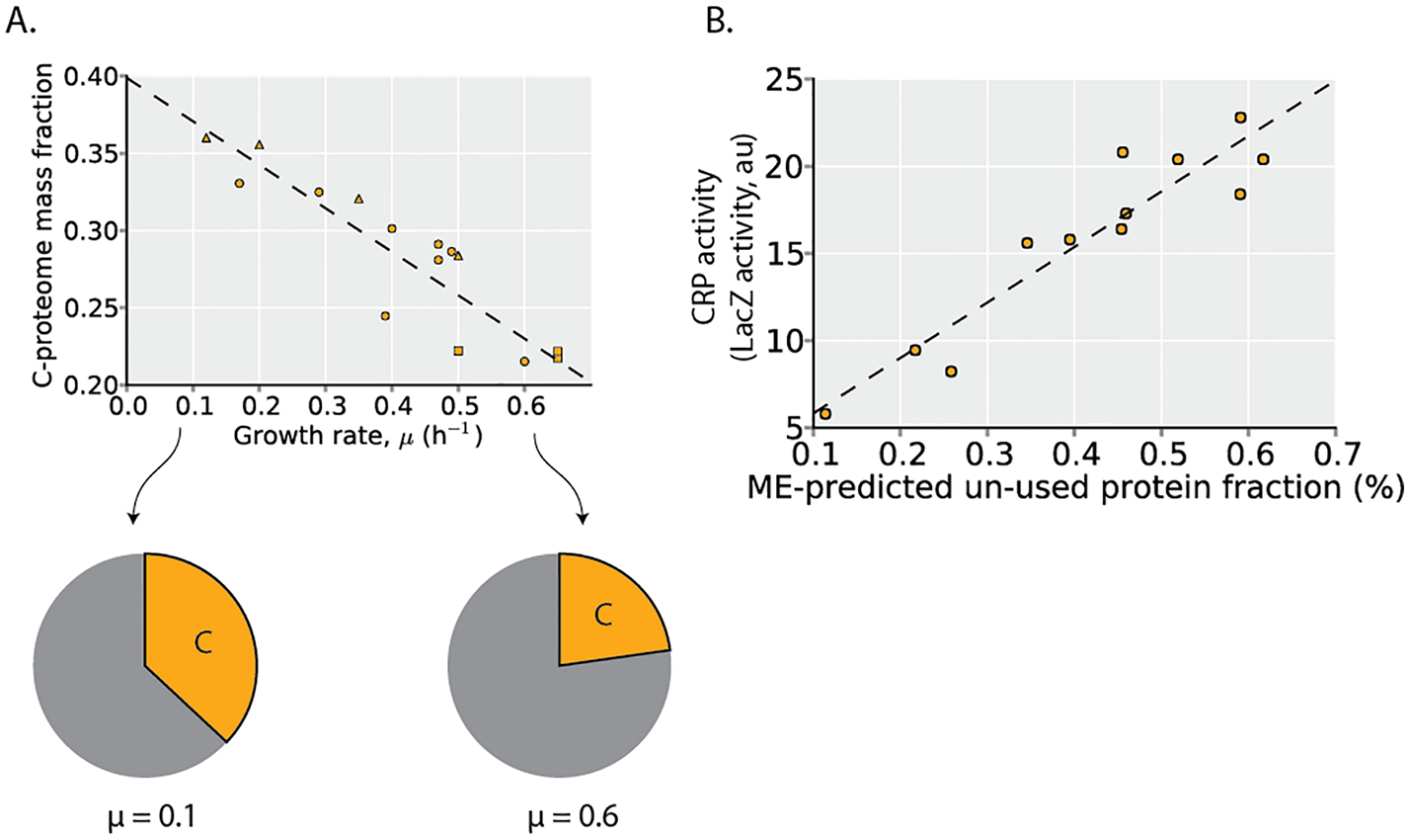
The non-core proteome is largely unutilized in a given environment. **(A)** The proteome mass fraction of the C-proteome is plotted as a function of growth rate in the profiled environments (which predominantly comprise carbon source shifts and carbon limitation). The dashed line is a linear regression (y = -0.28 x + 0.40, r^2^ = 0.81, p < 10^−4^). Point shape indicates environment type (carbon source batch cultures = circles, glucose-limited chemostat cultures = triangles, stress conditions = squares). The allocation to the C-proteome at different growth rates is shown in the pie charts based on the regression. (**B**) As the fraction of the proteome that is non-core and utilized remains relatively constant, the induction of non-core proteome segments by transcriptional regulators is expected to result in an increase in un-utilized protein. Using the ME-Model, the maximum un-used protein fraction under different carbon sources is computed based on measured growth rates from You et al. [23]. Measured Crp activity under different carbon sources from You et al. [23] is compared to the model-predicted un-used proteome fraction. The data is consistent with the hypothesis that Crp induces the expression of un-used protein.

The largest non-core proteome segment is the C-proteome (Fig 4A). In the conditions examined with quantitative proteomics, which predominantly comprise shifts and limitations in carbon sources, the C-proteome abundance decreases linearly with growth rate (Fig 6A). This linear relation with growth rate has been observed for individual proteins within the C-proteome proteome [23] (due to catabolite repression regulated by the transcription factor CRP) and serves to further validate the model-based definition of the C-proteome.

As previously shown, the non-core proteome (i.e., the C-, N-, P-, and S- proteome segments) is largely un-utilized. An important implication of this observation is that up-regulation of the non-core proteome segments largely results in increased expression of un-utilized protein (in the minimal media environments examined here). Across these environments profiled with proteomics (which predominantly comprise shifts and limitations in carbon sources), variation in the C-proteome abundance accounts for nearly all of the observed change in un-utilized protein (Fig 1C). Thus, up-regulation of the C-proteome under shifts in carbon sources and under carbon limitation, increases the expression of un-utilized protein.

The C-proteome is highly enriched in targets of the transcription factor CRP (Fig 4A), and CRP is known to up-regulate its target genes under growth limitation by carbon [23]. The change in abundance of the C-proteome under the conditions examined with proteomics may therefore be largely attributed to the action of CRP. We therefore hypothesized that higher CRP activity would result in higher un-utilized protein expression. To assess this hypothesis, we utilized CRP activity and growth rate data from You et al., measured from *E*. *coli* grown on 12 distinct carbon sources. The maximum unused protein fraction in each carbon source environment was calculated with the ME-Model while setting the growth rate to its measured values. Indeed, the model-predicted unused protein fraction is positively and linearly correlated with the measured CRP activity across environments (Fig 6B). These data and simulations further support the hypothesis that CRP activation largely results in the up-regulation of un-utilized protein in minimal media environments.

Thus, across these environments examined, changes in un-utilized protein can largely be attributed to the C-proteome and its regulators, including CRP. In other types of nutritional shifts (i.e., N, P, S limitations), however, the other identified proteome segments and regulators likely play a more predominant role. It may be that several of the global regulators identified as enriched in non-core proteome targets also largely result in the induction of un-utilized protein in minimal media environments.

### The non-ME proteome expression balances growth and stress resistance

**Fig 7:**
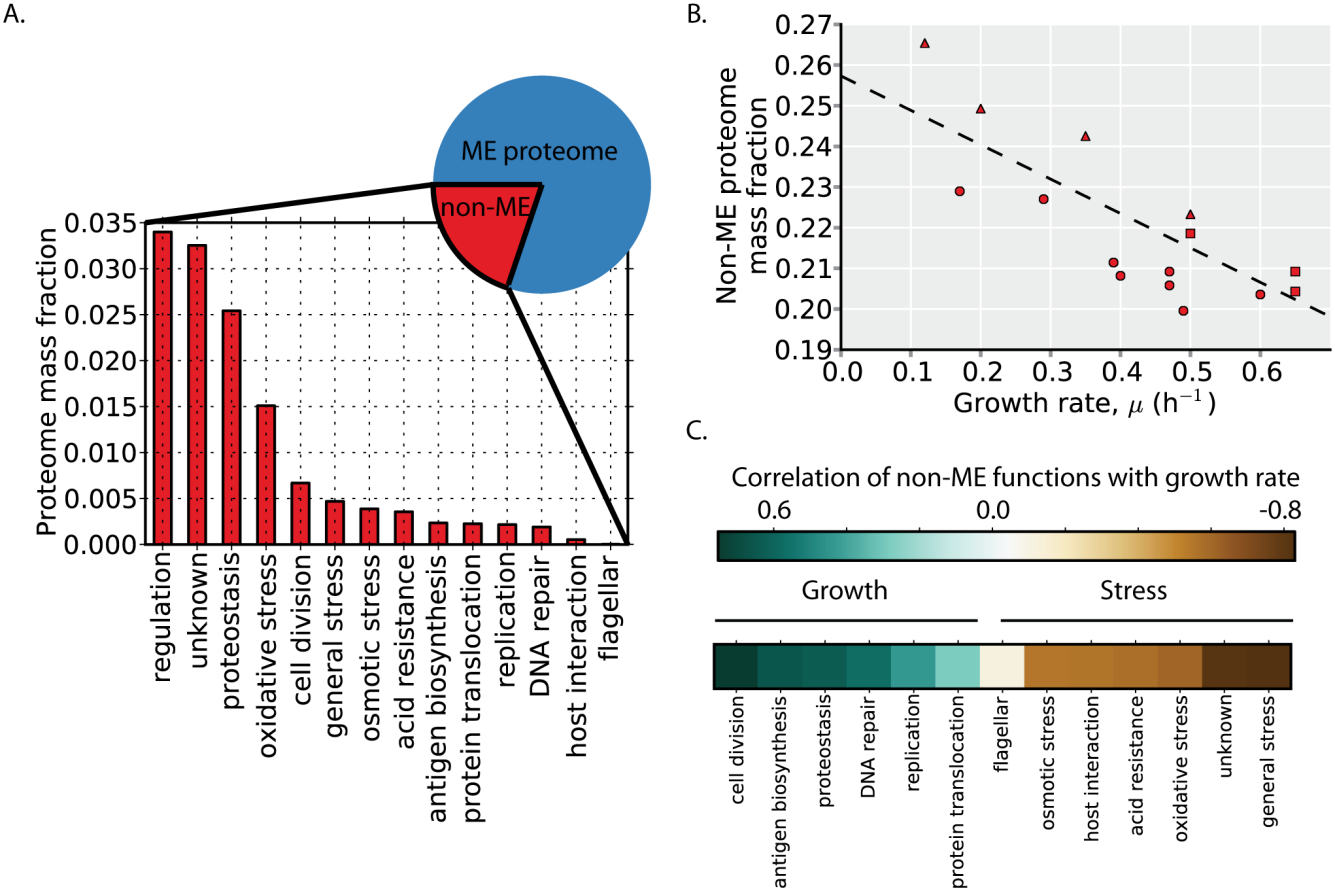
Growth versus stress regulatory logic. (**A**) The non-ME proteome encompasses ~20% of the proteome by mass in glucose minimal media (pie chart). The functional composition and abundance of the non-ME proteome (S1 Table) is shown based on proteomics data in glucose minimal media. (**B**) The proteome mass fraction of the non-ME proteome is plotted as a function of growth rate in the profiled environments. The dashed line is a linear regression (y = -0.09 x + 0.26, r^2^ = 0.53, p < 0.01). Point shape indicates environment type (carbon source batch cultures = circles, glucose-limited chemostat cultures = triangles, stress conditions = squares). (**C**) The median correlation of the proteins in each non-ME function is shown in the heatmap in rank order. While the overall non-ME proteome fraction is larger at higher growth rates, the trend depends on the specific function. Generally, functions positively correlated with growth rate (blue) are associated with biosynthetic and growth functions whereas functions negatively correlated with growth rate (brown) are associated with stress resistance.

Thus far, we have characterized only the proteome that is encompassed by the ME-Model (i.e., the ME proteome). We next examine the abundance, regulation, and function of the non-ME proteome. In glucose minimal media, the non-ME proteome comprises ~20% of the proteome by mass (Fig 7A, pie chart). The non-ME proteome allocation is not constant across environments, however, and overall, the non-ME proteome is slightly more abundant at lower than higher growth rates (Fig 7B).

To understand the function and regulation of the non-ME proteome, we first manually classify non-ME proteins by function using annotations and descriptions present in EcoCyc [24]. Much of the non-ME proteome mass can be classified by focusing on its most abundant proteins. We therefore classify the most abundant non-ME proteins that together comprise at least 80% of the non-ME proteome by mass across all 16 conditions examined (S1 Table). The abundant non-ME functions include replication, regulation, stress responses, and proteins of unknown function (encoded by so called y-genes). In glucose minimal media, the most abundant non-ME proteome functions are regulatory proteins and proteins of unknown function (Fig 7A).

While overall, the non-ME proteome is slightly more abundant at lower than higher growth rates, some non-ME proteome functions are positively correlated with growth rate, whereas others are negatively correlated with growth rate (Fig 7C). Broadly speaking, the functions that are positively correlated with growth rate are those related to growth, including cell division, replication, proteostasis, and protein translocation. The functions that are negatively correlated with growth rate are those related to stress resistance and survival, including osmotic, acid, and oxidative stresses. Thus, at higher growth rates, the growth-related functions are more highly expressed, and at lower growth rates, several stress resistance functions are more highly expressed.

## Discussion

### Microbial growth rates may be largely determined by unused protein expression

Since the first growth rate model by Monod [25], the quantitative determinants of microbial growth rates have been under investigation. As unused protein expression has been shown to have a causal role in decreasing growth rates in synthetic protein over-expression studies [4,5], microbial growth rates across different environments may largely be determined by unused protein expression as well. In support of this, we have shown that: 1) quantifying the cost of unused protein expression across several growth conditions explains the variability in growth rates across those environments (Fig 2), 2) strains selected for fast growth rates consistently decrease unused protein expression (Fig 3), and 3) the global regulator CRP predominantly regulates unused protein (across the minimal media environments examined) and the activity of the global regulator correlates with predicted unused protein expression levels (Fig 6). Thus, efforts to engineer strains for bioprocessing applications may benefit from directly eliminating highly expressed unused protein [6], utilizing adaptive evolution experiments to eliminate unused protein [18], or targeting transcription factors known to regulate unused protein [26].

While genome-scale models of metabolism are capable of accurate predictions of growth yields (gram dry weight per gram substrate) [27,28], predictions of growth rates by have only resulted in moderate correlations (Fig 2B) [7,29,30]. Our results suggest that unused protein expression is a key source of growth rate variability that has not been accounted for in genome-scale models to date and has limited the accuracy of growth rate predictions. Incorporation of the regulation of unused protein into genome-scale models may enable more accurate growth rate predictions [23,31].

We do not intend to imply that local rate-limiting bottlenecks in metabolic flux do not limit growth rates. Rather, these local bottlenecks and the global proteome allocation to unused protein can simultaneously limit growth rates; alleviation of either global or local bottlenecks can increase cellular growth rates. In fact, due to a cell’s native sensing and regulatory mechanisms, alleviation of local bottlenecks may naturally also result in reduction in unused protein expression (and vice versa). As an example, a mutation in glycerol kinase, glpK, alleviates a local rate-limiting step in glycerol metabolism during growth on glycerol [32]; this mutation also results in lower cAMP levels, which affects the expression of unused protein via its effect on the global transcriptional regulator CRP (Fig 3). On the other hand, a mutation in the RNA polymerase subunit rpoB (which can be considered a global regulator), results in the down-regulation of several un-utilized genes (mostly related to stress resistance) and the subsequent up-regulation of genes related to cellular growth [18]. Both mutations in global regulators and local rate-limiting bottlenecks are often found in adaptive evolution experiments [17,32–34], suggesting that alleviation of both local and global bottlenecks are viable mechanisms to increase cellular growth.

### Expression of an under-utilized core proteome may enable faster adaptation to improved growth conditions

While overexpression of an under-utilized core proteome incurs a fitness cost to steady-state growth (Fig 2D), the expression pattern of the core proteome (Fig 5) points to potential fitness benefits.

**Fig 8:**
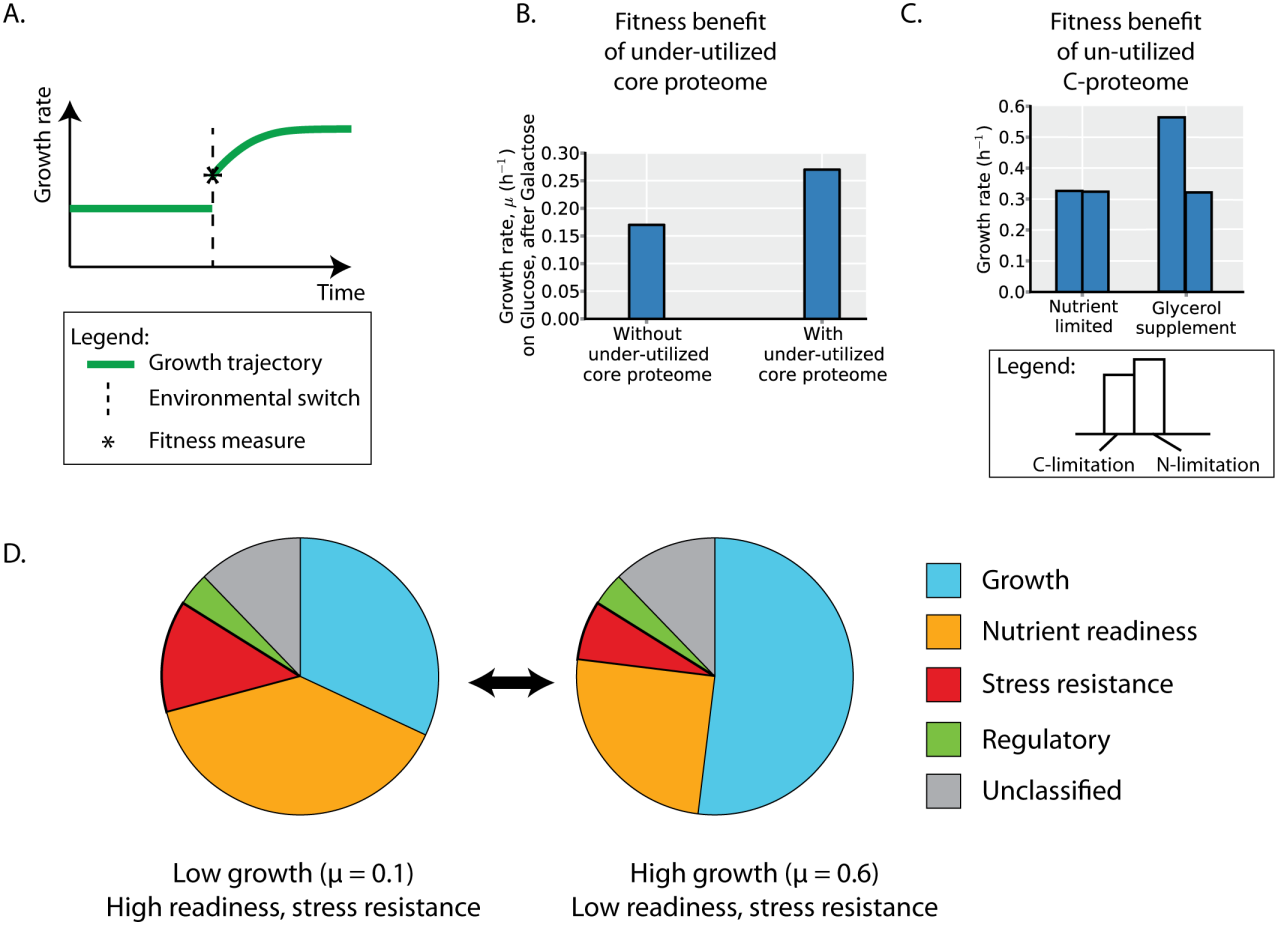
Fitness benefit of unused protein expression in changing environments results in fitness trade-offs. (**A**) Green line indicates organismal growth rate through an environmental shift. After an environmental shift, the instantaneous maximal growth rate is computed using the ME-Model (asterisk; which is limited by the expressed proteome prior to the shift) and is the measure of fitness used in B and C. (**B**) Expressing an under-utilized core proteome may provide a fitness benefit under changing environments. When shifting from galactose (the lowest growth carbon source profiled) to glucose (the highest growth carbon source profiled), having the core proteome initially in excess beyond the needs for biosynthesis enables a higher growth rate upon the nutrient up-shift (see Methods). (**C**) When carbon-limited, readiness for alternative carbon sources confers a fitness benefit whereas readiness for alternative nitrogen sources does not (see Methods). Up-regulation of the C-proteome under carbon limitation (but not nitrogen limitation) provides a fitness benefit. (**D**) The pie charts summarize the proteome allocation across the environments profiled. In environments with lower growth rates, the proteome allocated towards growth is lower, but the proteome allocated to nutrient readiness (the portions of the C-, N-, P-, and S- proteomes not utilized for growth) and stress resistance (portions of the non-ME proteome) is higher. As the proteome is a limited resource, proteome allocation to the different segments results in fitness trade-offs between growth, nutrient readiness, and stress resistance. Proteome segments are calculated based on regressions and proteome segments delineated in Figs 4–7.

The over-expression of the core proteome may enable a fitness benefit upon encountering more favorable growth environments that can support faster growth rates (Fig 8B). Rather than depending solely on *de novo* protein synthesis to meet the demands of faster growth, an over-expressed core proteome can enable faster adaptation. To demonstrate this effect, we simulate growth upon shifting from the lowest growth carbon source profiled (galactose) to the highest (glucose) with the ME-Model. The expressed proteome before the environmental shift is used to constrain the growth phenotype immediately following the shift (see Methods). If the core proteome is expressed in excess when growing in galactose, the organism grows faster upon the environmental ‘up-shift’; otherwise, the maximum instantaneous growth rate on glucose will be the same as that on galactose. This fitness benefit upon environmental up-shifts is consistent with experiments showing protein over-expression to incur a higher fitness cost upon environmental up-shifts (where the over-expressed core proteome becomes important for growth in the new environment) than down-shifts [35].

### Expression of an un-utilized C-proteome may balance resource allocation to growth versus nutrient readiness

While overexpression of the non-core (largely un-utilized) proteome segments incurs a fitness cost to steady-state growth (Fig 2C), the expression pattern of the C-proteome (Fig 6) points to potential fitness benefits.

The expression of the non-core proteome likely enables readiness for environmental change (Fig 8C). To demonstrate this effect, we simulate nutrient supplementations under C- and N-limitation with the ME-Model. Under C-limitation, supplementation with nitrogen sources provides no fitness benefit. On the other hand, supplementation with alternative sources of the limiting element provides growth advantages (Fig 8C). Thus, expression of the C-proteome provides a fitness benefit under C-limitation but not under N-limitation. While the non-core proteome segments are largely un-unutilized in a current environment, they may provide a fitness advantage upon environmental shifts. Indeed, higher activity of CRP has been shown to result in shorter lag phases at a cost of slower growth rates resulting in higher levels of catabolite repression to be beneficial in variable environments [36].

### Proteome allocation reflects fitness tradeoffs and ecological strategies

The previous sections suggest that the expression of unused protein can be attributed to proteome segments whose expression incurs a fitness cost on steady-state growth but also confers fitness benefits in varying environments.

Here, we summarize the model-defined proteome segments and their regulation with growth rate under variation in carbon source identity and availability. The proteome devoted to cellular growth in these minimal media environments is primarily composed of an invariant core proteome (Figs 4 and 5). Also contributing to growth is smaller portions of the non-core ME (Fig 4B) and non-ME proteomes (Fig 7C). The remaining non-core ME proteome can be considered to be devoted to nutrient readiness (Fig 6); these proteins can be utilized for growth when alternative nutrients become available. The non-ME proteome that is not devoted to cellular growth is predominantly related to stress functions (Fig 7C). Thus, quantifying the utilization and function of the proteome reveals its allocation towards growth, nutrient readiness, and stress resistance. At low growth rates, nutrient readiness and stress resistance functions are more abundant; at higher growth rates, allocation to these functions decreases to enable increased allocation to cellular growth (Fig 8D). Nutrient readiness and stress resistance functions can be considered ‘hedging’ functions that enable readiness for environmental change rather than being actively utilized, resulting in trade-offs between growth, nutrient readiness, and stress resistance due to proteome allocation constraints. Indeed, several studies have shown that slower growing cells are more resistant to cellular stresses [37–39].

An important implication of the identified fitness trade-offs is that cellular growth rates may be primarily determined by environmental history, rather than nutrient quality (i.e., the maximum growth rate possible with a specified nutrient, Fig 2A). As growth rates are determined by the proteome allocated towards growth, evolutionary and ecological factors may be more important than the identity and quality of the substrates themselves. For example, while glucose and galactose have similar inherent qualities as sole carbon sources (chemically and as defined by the growth potential of *E*. *coli* on these substrates), glucose is the highest growth substrate in this dataset and galactose is the lowest. Galactose may be a more rarely or transiently encountered carbon source or perhaps it is often associated with harsher environments, which would make environmental readiness and stress resistance comparatively more important components of overall cellular fitness [40–42]. The different regulatory patterns (e.g., different slopes and intercepts of the regressions in Figs 5A and 6A) observed across strains [4,23,43,44] may reflect and dictate their ecological strategy and environmental history and be balanced through the activity of global regulators [36,45].

### The proteome burden of a generalist species

While we broadly classify the proteome here based on nutrient readiness and stress resistance functions, it is important to realize that within these proteome segments is a variety of distinct functions enabling readiness for specific nutrients and stresses. Concerted up-regulation of these segments results in a general nutritional readiness and stress resistance. However, as an organism becomes prepared for a wider array of nutrients or stresses, these proteome segments must become larger. Therefore, in addition to a trade-off between the different components of organismal fitness identified here (growth, environmental readiness, stress resistance), proteome allocation constraints also result in a trade-off in specialist versus generalist ecological strategies. *E*. *coli* is a generalist species, capable of growing in a variety of environments. Its broad environmental niche results in large proteome burden. On the other hand, specialist species (capable of growing on a narrower range of substrates) would require a smaller proteome allocation to be equally ready for environmental change. Proteome allocation constraints will therefore also result in a trade-off between specialist and generalist strategies [46].

## Methods

### Proteomics dataset and normalization

The proteomics data was obtained from Schmidt et al. The dataset contains absolute protein counts per cell for *Escherichia coli* K-12 BW25113 grown in 16 different environments. The environments include batch culture with 8 different carbon sources (glucose, galactose, acetate, glycerol, glucosamine, fumarate, succinate, pyruvate) as the sole carbon substrate, 4 glucose-limited chemostat cultures (μ = 0.12, μ = 0.20, μ = 0.35, μ = 0.5), and 4 stress environments (high temperature [42°C], acid stress [pH 6], and osmotic stress [50 mM NaCl], anaerobic). Data from undefined rich media (LB) and stationary phase are not considered here as these environments cannot be readily simulated with the ME-Model. In total, global absolute abundance estimates are obtained for 2039 proteins across all conditions. All abundances are reported in copy number per cell. Growth rates from each environment are obtained from Volkmer et. al. [47]. For all analysis here, the protein copy numbers were transformed to mass fractions using the protein molecular weights.

### Quantifying the utilized and un-utilized proteome

To identify sets of proteins that can be utilized under a particular environment, we sample ME-Model enzymatic rate parameters; we then identify growth rate optimizing proteomes (for each parameter set) based on the growth-maximizing procedure outlined in O’Brien et al. [7]. We independently sampled all enzymatic rates in the ME-Model based on the global distribution of k_cat_ across all enzymes; we used a (base 10) lognormal distribution with mean μ = 1.11 and σ^2^ = 1.31, based on data from Bar-Even et al. [48]. All other model parameters are the same as described in O’Brien et al. [7].

For each carbon source present in the proteomics dataset, we simulated growth with 100 different sampled sets of enzymatic rates; these simulations result in 100 sets of proteins that are predicted to be utilized in that environment [19], and the abundance of these protein sets are interrogated in the proteomics data to obtain utilized and un-utilized proteome fractions (Fig 1B and 1C, S1 Fig).

### Quantifying the under-utilized proteome

For all proteins in the core proteome (see ME proteome classification), the ME-Model was used to predict the protein demand (for cell growth). Protein demand is defined as the protein abundance predicted by setting the growth rate to its measured value and maximizing the expression of an un-modeled protein. This procedure results in the minimal necessary expression of other proteins used for cell growth. Taking the ratio of the protein demand (i.e., model-predicted protein abundance) and the measured protein abundance, then gives a measure of the protein utilization. For each protein, to get the relative *in vivo* turnover for that protein (on a scale from 0 to 1), this ratio was then normalized by the maximum value (of the ratio) for that protein across all profiled environments (Fig 1D, S1 Fig).

### Growth rate predictions

Maximum growth rates are determined with the computational procedure described in O’Brien et al. [7]. Unused protein fraction and mean *in vivo* enzyme activity are changeable variables in the ME-Model that affect predicted growth rates. The values of these 2 variables inferred from the proteomics data (Fig 1) are set in the ME-Model to assess their effect on growth rates (Fig 2). The inferred un-utilized protein fraction (Fig 1C) is quantitative and directly set in the ME-Model. However, the inferred average *in vivo* enzyme activity (Fig 1D) is relative (on a scale from 0 to 1) and requires a quantitative value in one environment to determine the quantitative values from the other environments. This is accomplished by determining the average *in vivo* turnover that would result in the growth rate measured in glucose minimal media when the unutilized protein fraction is set to its inferred values (Fig 1C) and all other model parameters are the same as described in O’Brien et al. [7]. (S2 Fig). When the unused protein fraction and mean *in vivo* enzyme are kept constant across all environments (Fig 2A), they are set to the values inferred from glucose batch culture. All other model parameters are the same as described in O’Brien et al. [7].

### Quantifying un-utilized and under-utilized transcriptome from adaptive evolution experiments

These values are computed the same way as the un-utilized and under-utilized proteome fraction (Fig 1; see “Quantifying the utilized and un-utilized proteome” and “Quantifying the under-utilized proteome”). Rather than using proteomics data, however, transcriptomics data is used. The protein-coding gene’s transcriptome fraction was estimated using its gene length and expression level measured in FPKM obtained from cufflinks and provided in LaCroix et al. A gene’s transcriptome fraction was taken to be the product of FPKM and the gene length, divided by the sum of this product over all genes. The utilized transcriptome fraction was then calculated by summing the transcriptome fractions of all utilized genes. The utilized transcritome fraction is computed for all sets of utilized genes found by sampling enzymatic parameters (see “Quantifying the utilized and un-utilized proteome”), resulting in a distribution of utilized transcriptome fraction values.

### ME proteome classification

All growth-supporting minimal media were simulated with the ME-Model. The minimal media were defined by starting from the default glucose M9 medium (with ammonium as a nitrogen source, phosphate as a phosphorus source, and sulfate as a sulfur source) and by individually changing all carbon, nitrogen, phosphorous, and sulfur sources. In total, 333 environments were simulated, corresponding to 180 carbon, 93 nitrogen, 49 phosphorus, and 11 sulfur sources. Isozymes were required to be used in equal abundance. All expressed proteins were identified as those with non-zero translation fluxes. Proteins expressed across all simulated environments were considered the core proteome. The difference in the definition of the core proteome provided here and that of Yang et al. is that isozymes are required to be used in equal abundance. Proteins not in the core, but expressed under certain alternative Carbon, Nitrogen, Phosphorous, or Sulfur sources are considered in the C-, N-, P-, and S-proteome segments, respectively. A protein may belong to more than one non-core proteome segment (S3 Fig). All other model parameters are the same as described in O’Brien et al. [7].

### Fitness benefit simulations for the under-utilized core proteome

Steady-state growth was first simulated with the ME-Model in the initial environment (galactose batch culture). Two scenarios were considered: one in which the *in vivo* enzyme turnover was equal to that measured in the proteomics data in galactose batch culture and the other in which the *in vivo* enzyme turnover was equal to that measured in the second environment (glucose batch culture). The predicted protein abundance to support growth for all proteins in the core proteome were obtained from the simulation output. Then, the maximal growth rate after an environmental shift (to glucose batch culture) was computed subject to the expression level of the core proteome expression prior to the shift for both scenarios.

### Fitness benefit simulations for the non-core ME proteome

The uptake rate of glucose and ammonium was limited in glucose minimal media to simulate carbon (C-) and nitrogen (N-) limited growth by limiting the uptake reaction flux. Then, subject to the glucose and ammonium uptake limitations, additional carbon (glycerol) sources were supplied in excess and maximal growth rates predicted.

### non-ME proteome classification

A subset of the proteins outside of the scope of the ME-Model (i.e., the non-ME proteome) was manually classified by function. For each environment, the most abundant proteins, comprising at least 80% of the non-ME proteome mass were annotated based on descriptions from EcoCyc [24].

## Supporting Information

S1 Fig Computational method to identify the utilized proteome and relative *in vivo* enzyme turnover. (**A**) There is more than one set of proteins that can support growth under a given environment. This uncertainty is accounted for in the depicted procedure that combines both model simulations and measured proteomics data (see Methods). First, the sets of proteins that can support growth in the specified environment are enumerated by the ME-Model through sampling different model parameters (enzyme activities), each resulting in protein expression vectors. Each expression vector then defines a utilized protein set, and the total abundance (proteome mass fraction) of these proteins is determined in the proteomics dataset. Calculating the expressed proteome mass fraction of all utilized protein sets results in a distribution for the utilized proteome fraction. (**B**) The distributions of relative *in vivo* enzyme turnover across all proteins in the core ME proteome are shown, with boxplots plotted arranged according to the growth rate in that environment. Red dots and line indicate median values, and boxes indicate quartiles; outliers (crosses) are considered greater than 1.5 times the interquartile range.
(TIF)

S2 Fig Model parameterization of quantitative average *in vivo* enzyme turnover to predict growth rates. To predict growth rates with the ME-Model (Fig 2), average in vivo enzyme activities must account for changes the under-utilized proteome (Fig 1D). The inferred *in vivo* enzyme activity (Fig 1D) is relative (on a scale from 0 to 1) and requires a quantitative value in one environment to determine the quantitative values from the other environments. To accomplish this, the average *in vivo* enzyme turnover in glucose minimal media is inferred based on the measured growth rate (blue circle). The un-used proteome fraction is set to the level inferred in Fig 1C. All other model parameters are as defined in O’Brien et al. Dotted line is a linear regression based on ME-Model computed maximum growth rates (red squares).
(TIF)

S3 Fig The relationship between change in ribosomal protein transcriptome fraction and enzyme turnover in experimentally evolved strains. To corroborate the inferred change in *in vivo* enzyme turnover rates in experimentally evolved strains from LaCroix et al. (Fig 3B), we compare the change in *in vivo* enzyme turnover to the change in the ribosomal protein transcriptome fraction. As all of the evolved strains have similar growth rates, a lower ribosomal protein transcriptome fraction implies a higher translation rate (amino acids per ribosome per second). Consistent with this, the change ribosomal protein fraction is negatively correlated with the change in *in vivo* enzyme turnover. One strain (strain 8) actually decreases the expression of ribosomal proteins compared to the wild-type strain even though the evolved strain’s growth rate is ~1.0 h^-1^ compared to 0.7 h^-1^ in the wild-type, suggesting that translation rates are higher in the evolved strains.
(TIF)

S4 Fig Core and non-core (conditionally-utilized) proteome composition and abundance. Shown is the functional composition of the ME-Model-defined core proteome (**A**) and conditionally-utilized (non-core) proteome (**B**), based on KEGG annotations. Visualization was created using Proteomaps (www.proteomaps.net). (**C**) Overlap of proteins in the conditionally-utilized ME proteome sectors is shown in the 4-way Venn diagram.
(TIF)

S5 Fig Non-ME proteome composition and abundance. Shown is the functional composition of the non-ME proteome, based on KEGG annotations. The large ‘Not mapped and “other enzymes’ indicate an incomplete functional annotation of protein comprising the proteome outside of the ME-Model. Areas are proportional to abundances based on the measured expression levels in glucose minimal media. Visualization was created using Proteomaps (www.proteomaps.net).
(TIF)

S1 Table ME and non-ME proteome segments. (XLSX)
