# Supplementary figures and images for: Quantification and Classification of E. coli Proteome Utilization and Unused Protein Costs across Environments
Source: PLoS Comput Biol. 2016 Jun 28;12(6):e1004998. doi: 10.1371/journal.pcbi.1004998 (PMC4924638; doi:10.1371/journal.pcbi.1004998)

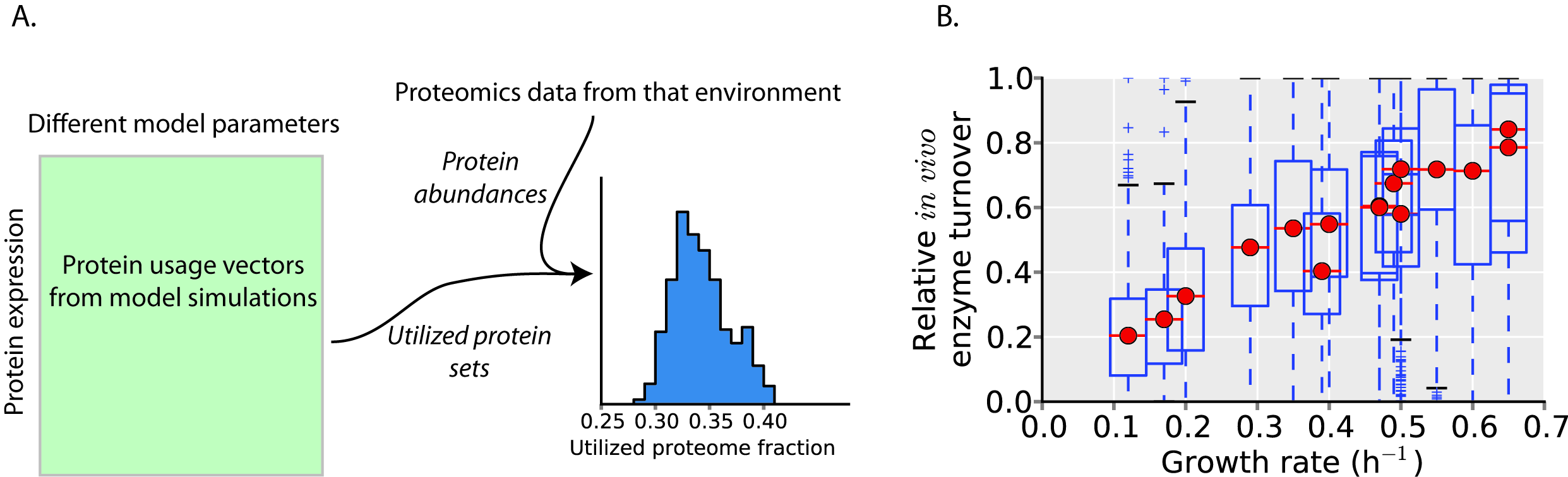

Supplement: S1 Fig — (A) There is more than one set of proteins that can support growth under a given environment. This uncertainty is accounted for in the depicted procedure that combines both model simulations and measured proteomics data (see Methods). First, the sets of proteins that can support growth in the specified environment are enumerated by the ME-Model through sampling different model parameters (enzyme activities), each resulting in protein expression vectors. Each expression vector then defines a utilized protein set, and the total abundance (proteome mass fraction) of these proteins is determined in the proteomics dataset. Calculating the expressed proteome mass fraction of all utilized protein sets results in a distribution for the utilized proteome fraction. (B) The distributions of relative in vivo enzyme turnover across all proteins in the core ME proteome are shown, with boxplots plotted arranged according to the growth rate in that environment. Red dots and line indicate median values, and boxes indicate quartiles; outliers (crosses) are considered greater than 1.5 times the interquartile range. (TIF) [file pcbi.1004998.s001.tif]

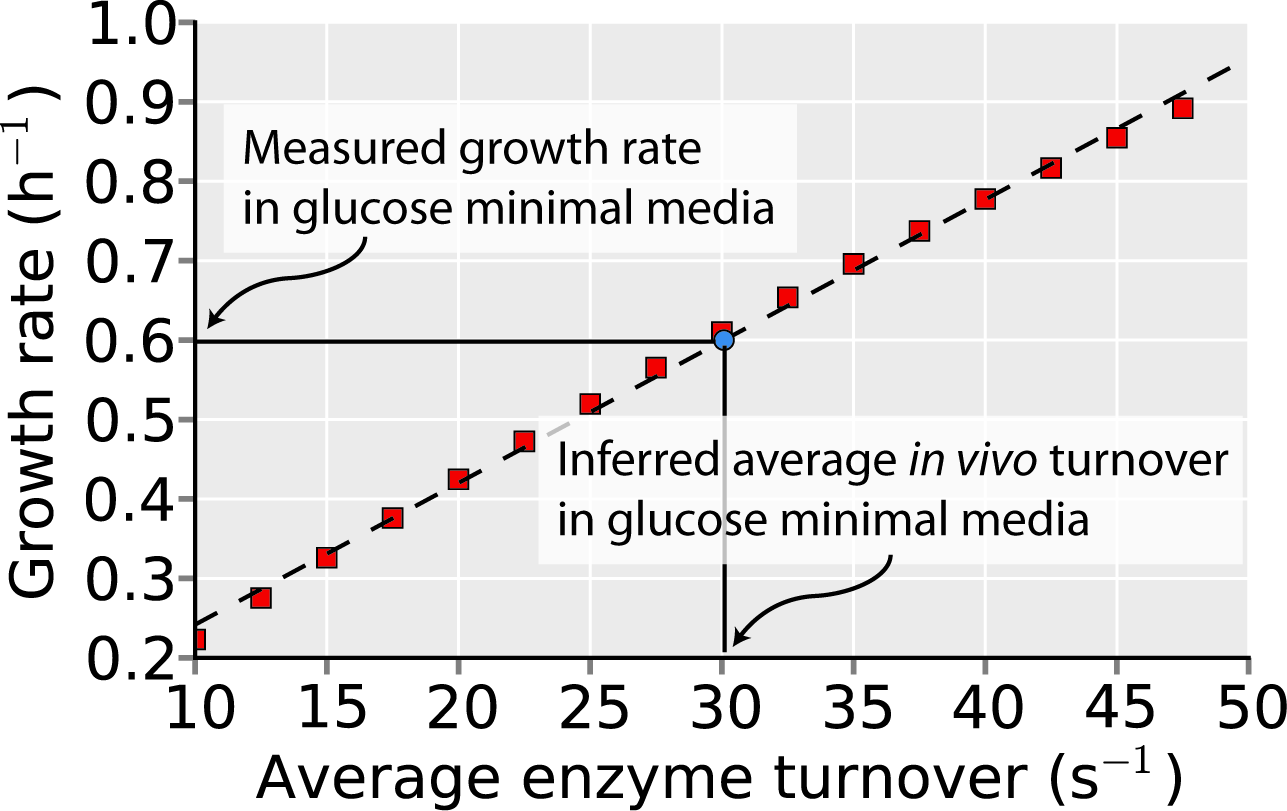

Supplement: S2 Fig — To predict growth rates with the ME-Model (Fig 2), average in vivo enzyme activities must account for changes the under-utilized proteome (Fig 1D). The inferred in vivo enzyme activity (Fig 1D) is relative (on a scale from 0 to 1) and requires a quantitative value in one environment to determine the quantitative values from the other environments. To accomplish this, the average in vivo enzyme turnover in glucose minimal media is inferred based on the measured growth rate (blue circle). The un-used proteome fraction is set to the level inferred in Fig 1C. All other model parameters are as defined in O’Brien et al. Dotted line is a linear regression based on ME-Model computed maximum growth rates (red squares). (TIF) [file pcbi.1004998.s002.tif]

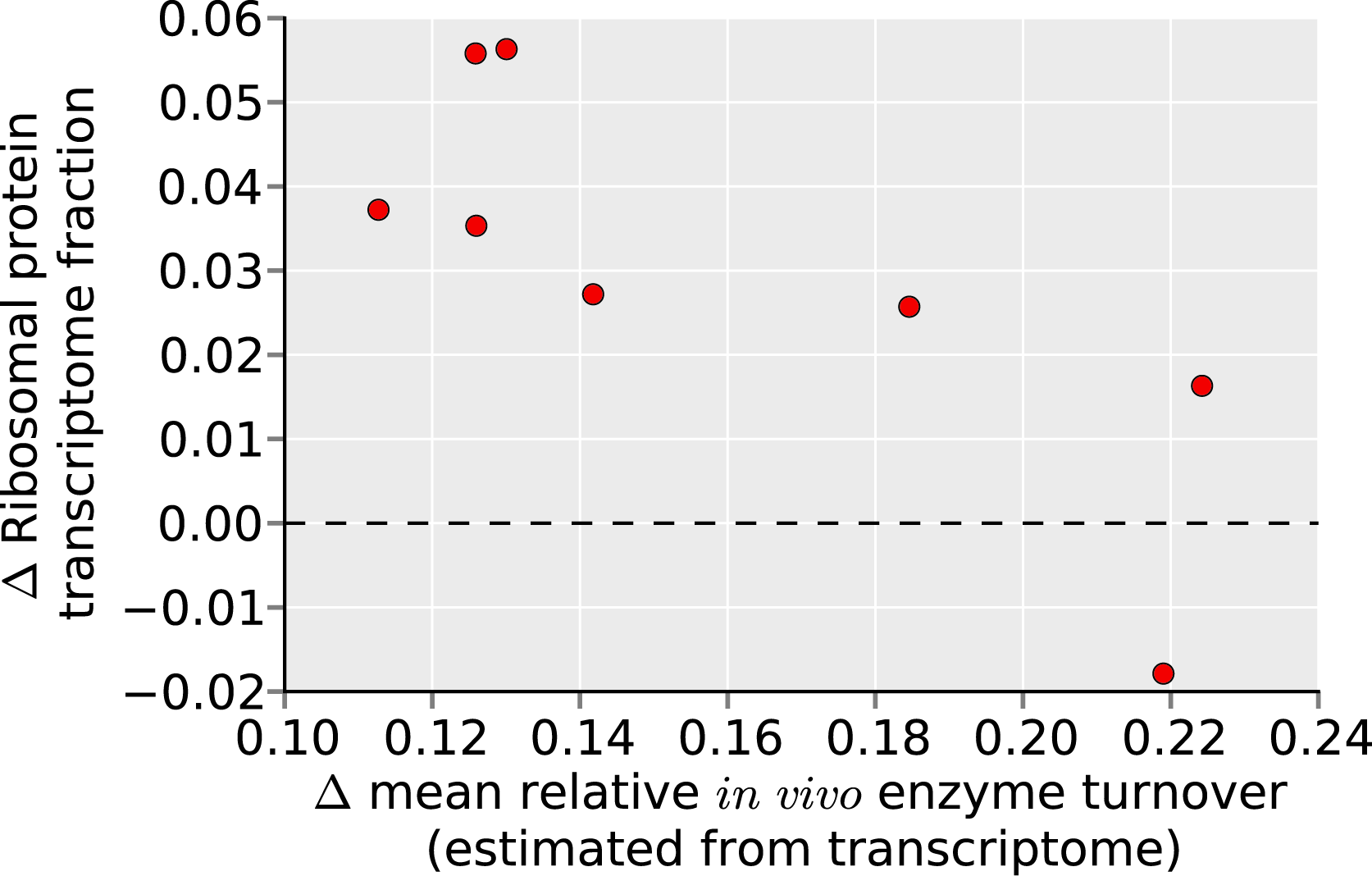

Supplement: S3 Fig — To corroborate the inferred change in in vivo enzyme turnover rates in experimentally evolved strains from LaCroix et al. (Fig 3B), we compare the change in in vivo enzyme turnover to the change in the ribosomal protein transcriptome fraction. As all of the evolved strains have similar growth rates, a lower ribosomal protein transcriptome fraction implies a higher translation rate (amino acids per ribosome per second). Consistent with this, the change ribosomal protein fraction is negatively correlated with the change in in vivo enzyme turnover. One strain (strain 8) actually decreases the expression of ribosomal proteins compared to the wild-type strain even though the evolved strain’s growth rate is ~1.0 h-1 compared to 0.7 h-1 in the wild-type, suggesting that translation rates are higher in the evolved strains. (TIF) [file pcbi.1004998.s003.tif]

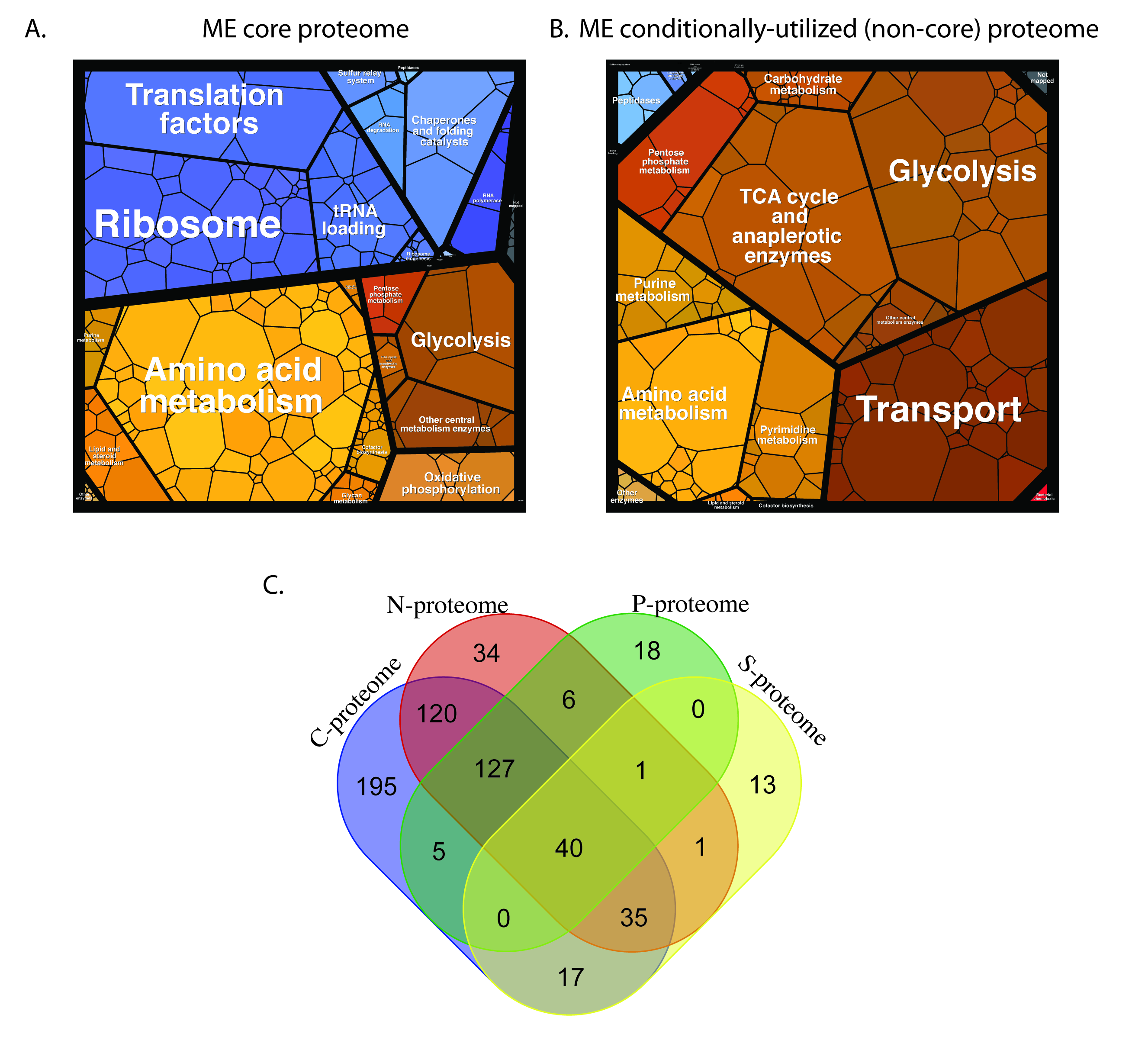

Supplement: S4 Fig — Shown is the functional composition of the ME-Model-defined core proteome (A) and conditionally-utilized (non-core) proteome (B), based on KEGG annotations. Visualization was created using Proteomaps (www.proteomaps.net). (C) Overlap of proteins in the conditionally-utilized ME proteome sectors is shown in the 4-way Venn diagram. (TIF) [file pcbi.1004998.s004.tif]

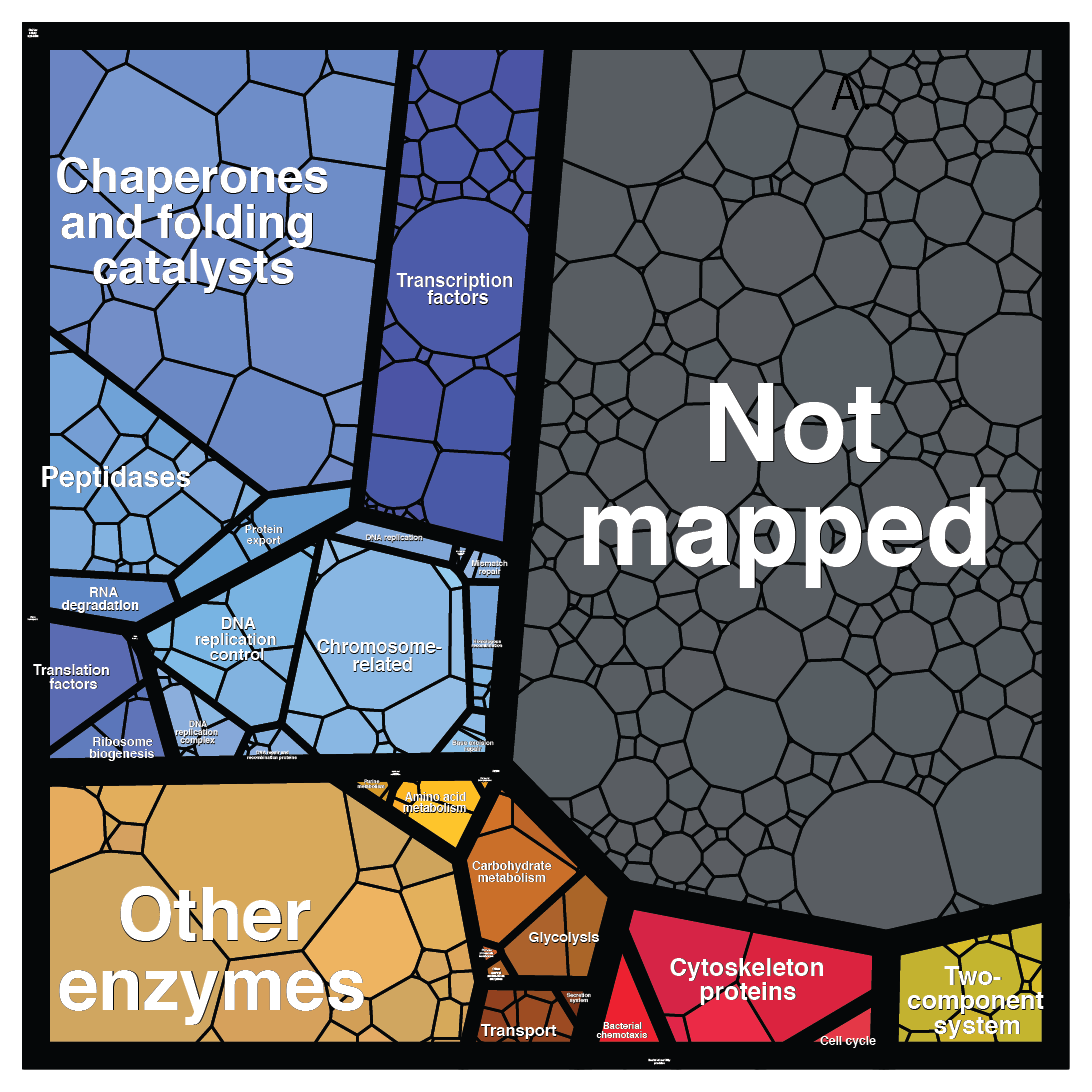

Supplement: S5 Fig — Shown is the functional composition of the non-ME proteome, based on KEGG annotations. The large ‘Not mapped and “other enzymes’ indicate an incomplete functional annotation of protein comprising the proteome outside of the ME-Model. Areas are proportional to abundances based on the measured expression levels in glucose minimal media. Visualization was created using Proteomaps (www.proteomaps.net). (TIF) [file pcbi.1004998.s005.tif]
